# Retraction: MicroRNA-326 functions as a tumor suppressor in breast cancer by targeting ErbB/PI3K signaling pathway

**DOI:** 10.3389/fonc.2023.1298871

**Published:** 2023-10-10

**Authors:** Frontiers Editorial Office

Following publication, concerns were raised regarding the validity of the data in the article. The authors failed to provide the raw data and a satisfactory explanation during the investigation, which was conducted in accordance with Frontiers’ policies. Given the concerns about the validity of the data, and the lack of raw data, the editors no longer have confidence in the findings presented in the article. This retraction was approved by the Chief Editors of Frontiers in Oncology.

The authors did not agree with the retraction.

